# Stressors and coping styles of nursing students in the middle period of clinical practicum: a qualitative study

**DOI:** 10.1186/s12912-024-02063-z

**Published:** 2024-06-07

**Authors:** Fengzhen Li, Yawei Zeng, Yingjie Fu, Yuenv Wang, Tingting Lin, Qianying Deng, Jufang Li

**Affiliations:** School of Nursing, Wenzhou Medical University, Wenzhou, Zhejiang China

**Keywords:** Nursing students, Stressors, Coping styles, Middle period of clinical practicum

## Abstract

**Background:**

Nursing students encounter various stressors during their clinical practicum; however, the stressors are not the same during different periods. At present, studies on the stressors and coping styles of nursing students in the middle period of their clinical practicum are rare.

**Aims:**

The current study aimed to explore the stressors and coping styles of nursing students in the middle period of their clinical practicum.

**Methods:**

A qualitative study with a descriptive phenomenological method was conducted to collect data from 10 nursing students undergoing the middle period of their clinical practicum from December 2020 to February 2021. The data were collected by semistructured interviews using interview outlines prepared in advance. The data were analyzed by Colaizzi’s analysis method.

**Results:**

The stressors experienced by nursing students in the middle period of their clinical practicum mainly included personal reasons, teaching arrangements, interpersonal relationships, occupational particularity and career planning. Additionally, nursing students coped with the stressors that they face in the clinical practicum by eliminating stressors and regulating emotions.

**Conclusions:**

Nursing students experienced various stressors and used a variety of coping styles in the middle period of their clinical practicum, which was different from what occurred in the early and late periods. Targeted interventions should be formulated and implemented to relieve nursing students’ stress and guide them to adopt effective coping styles.

## Introduction

The clinical practicum is the last period of nursing education in China. Nursing students can develop basic practical, critical thinking and communication skills through the clinical practicum that can help them make the transition to professional nurses [1]. However, the clinical practicum is considered a major source of stress in nursing education [2] and may cause physiological and psychological problems such as anxiety, insomnia and inattention [3]. The abovementioned problems can reduce clinical nursing competence and even cause burnout and low levels of job satisfaction in nursing students [4]. As a common practice in China, nursing students are trained through a continuous 10-month clinical practicum. For the 10-month clinical practicum, nursing students are required to go through rotations in 10 departments, namely internal medicine (2 departments), surgery (2 departments), obstetrics and gynecology (1 department), pediatrics (1 department), emergency medicine (1 department), intensive care unit (1 department), operating room (1 department) and a special department (with an outstanding team of experts and technology). The rotation cycle of each department lasts 4 weeks. Then, the students need to go through a 2-week community practicum and a 1-week psychiatric practicum. As nursing students behave differently in the different periods, scholars have divided the 10-month clinical practicum into early (the first three months), middle (the fourth to seventh month), and late (the eighth to tenth month) periods [5]. Studies have shown that the stressors and coping styles of nursing students vary during different periods [6]. In the middle period of the clinical practicum, most nursing students have adapted to the clinical environment. During this period, nursing students are eager to improve their technical skills, and their need for respect and self-realization is prominent [5]. Therefore, the middle period of the clinical practicum is the key time for cultivating high-quality nursing students, and understanding the stressors and coping styles of nursing students at this stage is particularly important. However, studies on the stressors and coping styles of nursing students have focused mainly on the early or late period of their clinical practicum [1, 7, 8] and little is known about the stressors and coping styles of nursing students in the middle period of their clinical practicum. Therefore, understanding the stressors and coping styles of nursing students in the middle period of their clinical practicum is crucial for improving the quality of nursing and promoting their psychosomatic health.

Stress refers to a situation that occurs when an individual’s internal or external demands are perceived as consuming or exceeding his or her own adaptation or coping resources [9]. The stress experienced by nursing students during their clinical practicum is seen as the difference between the needs of nursing students in a given clinical situation and their resources or ability to perform tasks [2]. Moderate stress can stimulate the potential and learning desire of nursing students, while long-term or excessive stress has a negative impact on the psychosomatic state of nursing students and decreases their performance in their clinical practicum [10, 11]. Clinical issues, academic issues, employment prospects, financial and health issues are the key stressors for nursing students [12–18]. Studies have shown that nursing students have the highest stress level in the early period of their clinical practicum [2]. Among these students, lack of professional knowledge or clinical skills, interpersonal relationships, clinical environment, patient care and workload are considered common stressors for nursing students during this period [19, 20]. In addition, nursing students’ stress persists through the late period of their clinical practicum [21]. Approximately 85.3% of nursing students experience a moderate to high level of stress in the late period of their clinical practicum [8], and employment prospects and graduation examinations are the main stressors they face during this period [7]. However, few studies have been conducted to explore the stressors of nursing students in the middle period of their clinical practicum.

Coping style is the continuous effort to overcome the unbalanced conditions caused by internal and external demands [9]. The use of effective coping styles helps students regain a state of balance, thereby reducing the negative effects of stress. Nevertheless, improper coping styles increase the negative impact of stress [22], which in turn affects the clinical practicum performance of nursing students. Solving problems, transference, staying optimistic and avoiding problems are common coping styles used by nursing students [23]. Studies have shown that nursing students’ coping styles dynamically change [24]. In the early period of clinical practicum, changing cognition, talking and comforting oneself are the most common coping strategies used by nursing students [1]. In the late period of clinical practicum, nursing students have higher scores for positive coping styles than for negative coping styles [8, 25]. In addition, some nursing students have expressed that talking, comforting oneself and avoidance are the most common coping styles [26]. At present, studies on the coping styles of nursing students in the middle period of their clinical practicum are rare; thus, further exploration is needed.

In summary, nursing students encounter a variety of stressors during their clinical practicum, and they adopt various coping styles in the face of stress. Additionally, the stressors and coping styles of nursing students vary during different periods [6]. However, the current research on nursing students’ stressors and coping styles has focused mainly on the early and late periods of clinical practicum; few studies have explored the stressors and coping styles of nursing students in the middle period of their clinical practicum. Therefore, the descriptive phenomenological method was used in this study to explore in depth the stressors and coping styles of nursing students in the middle period of their clinical practicum to provide a basis for nursing educators to formulate corresponding intervention strategies.

## Materials and methods

### Design

A qualitative study with the descriptive phenomenological method was used to explore the stressors and coping styles of nursing students in the middle period of their clinical practicum. Descriptive phenomenology is a method based on Husserl’s philosophy, which advocates “returning to the thing itself”. From this point of view, researchers need to adopt an attitude of “getting out of the way” to conduct research and provide unbiased and pure descriptions of phenomena [27]. The purpose of the descriptive phenomenological method is to describe the nature of a meaningful experience or its essential structure [28], which can be effective in helping us explore the stressors and coping styles of nursing students in the middle period of clinical practicum.

### Setting and sample

The population for the qualitative study included nursing students who were interns at a third-class hospital in China. Currently, Chinese nursing students are trained at medical universities for four years. Specifically, for the first three years, they are trained in theoretical study at the university, while for the fourth year, they are trained at hospitals affiliated with the medical university for their clinical practicum. Purposive sampling was used to select nursing students. Purposive sampling involves the extraction of people, places and events that can provide the most information for the research problem [29]. Based on the purpose of the study, we published recruitment information in the internship WeChat group and recruited eligible nursing students in the middle period of their clinical practicum as interviewees.

The inclusion criteria for the interviewees were as follows: (1) were in the middle period of their clinical practicum and (2) volunteered to participate in this study. We did not have exclusion criteria because all the nursing students had a high level of literacy and were able to express themselves clearly. A total of 10 nursing students were included in this study and none dropped out.

### Instruments

Based on a literature review [7, 11, 24] and the purpose of the study, the interview outline was designed as follows: (1) Do you currently feel any stress during your clinical practicum? What do you find stressful? Can you give me an example? (2) Do you need to add something about the stressors you have encountered in your clinical practicum? (3) How do you normally deal with this stress? Please give an example. (4) What help do you need for these problems that you have encountered during your clinical practicum?

### Data collection

The data were collected through face-to-face interviews held from December 2020 to February 2021. All the authors are female graduate students except the corresponding author, who is a female doctor. The interview outline was designed by the research team. The qualitative interviews were conducted by the first and second authors, who have taken qualitative research courses and who have a deep understanding of qualitative research. The interviewees had no previous knowledge of the qualitative researchers. The qualitative researchers interviewed one interviewee at a time, and there were no repeated interviews. The interview location was an empty classroom to ensure a private and quiet environment, and there was no one else present besides the interviewee and researchers. Before the interviews, the interviewees needed to complete general information questionnaires. During the interviews, open-ended questions were used to explore the feelings of the interviewees in detail, but guidance and suggestive language were avoided. The interviews lasted between 20 and 30 min each, and the whole process was recorded by a voice recorder. The interviewees’ emotions, manners and other nonverbal behaviors were also recorded in a notebook. The interview records and notes were analyzed on the day of the interview, and the data were considered saturated when no new themes or subthemes appeared in the data analysis. The transcripts were returned to the participants for commenting and/or correction. The data were reanalyzed if there was any deviation.

### Data analysis

The data were analyzed by the first and second authors. NVivo 12 was used to encode and classify the data. Colaizzi’s seven-step analysis method [30] was used for analysis: (1) read each text carefully and repeatedly to understand all the materials; (2) read the text word by word and underline important words and sentences; (3) construct/encode the meaning of recurring ideas; (4) collect the encode views to find meaningful common concepts and form the rudiment of the themes; (5) define and describe each prototype topic and extract some typical original statements and insert them into each topic description; (6) put together similar prototypes and their descriptions for repeated comparison and identify and extract similar views to form the theme; and (7) return the generated topic structure to the research participants for verification.

### Rigor

Three methods were used to ensure the rigor of this qualitative study. First, a purposive sampling strategy was used to maximize the range of relevant information. Second, the audio-recorded interviews were transcribed verbatim to ensure the accurate description of the students’ experiences. Finally, the data were verified with the participants to ensure that the interpretations were free from bias.

## Results

### Characteristics of participants

As shown in Table 1, the ten participants were all female (100%). Six students (60%) were 22 years old, and eight (80%) were from rural areas. In addition, six students (60%) were only child and chose nursing involuntarily. Only three students (30%) were preparing for the postgraduate entrance examination. In addition, seven students (70%) expressed their willingness to become clinical nurses.

**Table 1:** Demographic characteristics of the participants (*N* = 10)

Participants	Sex	Age	Residential area	Only child	Choosing nursing voluntarily	Preparing for postgraduate entrance examination	Willing to be clinical nursing staff
N1	Woman	22	Urban	No	Yes	No	Yes
N2	Woman	23	Rural	Yes	Yes	Yes	Yes
N3	Woman	23	Rural	Yes	Yes	No	Yes
N4	Woman	23	Rural	No	No	No	Yes
N5	Woman	23	Rural	Yes	No	No	No
N6	Woman	22	Rural	No	No	No	Yes
N7	Woman	22	Rural	Yes	Yes	No	No
N8	Woman	22	Rural	Yes	No	Yes	Yes
N9	Woman	22	Rural	No	No	No	Yes
N10	Woman	22	Urban	Yes	No	Yes	No

### The themes and subthemes of stressors

Five stressor themes were identified in this study: personal reasons, teaching arrangements, interpersonal relationships, occupational particularity and career planning (Table 2).

**Table 2:** The themes and subthemes of stressors

Themes	Subthemes	Number of students	Rank
Personal reasons	A. Financial stress	2 (N5, N7)	13
	B. Physical fitness	2 (N5, N9)	13
Teaching arrangements	A. Departmental rotation	5 (N3, N4, N8, N9, N10)	5
	B. Departmental examination	4 (N3, N6, N7, N10)	9
	C. Nurse certification examination	4 (N3, N4, N5, N6)	9
	D. Graduation thesis	2 (N4, N5)	13
	E. School theory examination	2 (N2, N9)	13
Interpersonal relationships	A. Nurse‒patient relationships	10 (N1, N2, N3, N4, N5, N6, N7, N8, N9, N10)	1
	B. Teacher‒student relationships	5 (N4, N5, N6, N7, N9)	5
	C. Classmate relationships	2 (N4, N7)	13
	D. Doctor‒nurse relationships	2 (N3, N5)	13
Occupational particularity	A. Work intensity	4 (N1, N2, N7, N10)	9
	B. Patient safety	6 (N1, N2, N4, N5, N8, N9)	2
	C. Specialized operation	5 (N3, N5, N6, N7, N8)	5
	D. Occupational exposure	6 (N2, N4, N5, N8, N9, N10)	2
	E. Scheduling system	6 (N2, N4, N5, N7, N9, N10)	2
Career planning	A. Employment	5 (N3, N4, N6, N7, N8)	5
	B. Further education	3 (N6, N8, N10)	12

#### Personal reasons

The personal reasons included two subthemes: financial stress and physical fitness.

##### Financial stress

The nursing students receive no salary during their clinical practicum, which causes financial stress for some students. In this study, two nursing students reported financial stress.

“Other professional internships are paid every month, while our major has no salary; so I have to rely on my family for financial support. I feel quite stressed about this.” (N5).

“We work for such a long time on the clinical practicum and have to rely on our family to provide financial support, so it is really stressful.” (N7).

##### Physical fitness

Physical discomfort affects nursing work and can indirectly pressure nursing students. In this study, two nursing students were stressed about physical fitness.

“A few days ago, I caught a cold and runny nose at work, which affected my work. Later, when it was serious, my hearing level suddenly decreased. I couldn’t hear what a patient said clearly, so I asked him to repeat it again and again. Because of this, there was a little pressure that day.” (N5).

“During this time, I asked for leave because I was sick, so I felt that I was not helpful to my teacher. I also felt a little bit pressure because my teacher was always concerned about me.” (N9).

#### Teaching arrangements

The teaching arrangement included five subthemes: departmental rotation, departmental examination, nurse certification examination, graduation thesis and school theory examination.

##### Departmental rotation

The nursing students expressed inadaptability when entering a new department, which was mostly related to changes in the environment and unfamiliarity with the theoretical knowledge and equipment used by the new department. In this study, five nursing students were stressed about their departmental rotations.

“Every time to a new department, I am unfamiliar with the new equipment or technology at the beginning, so I feel very flustered during the process.” (N8).

“Every time I go to a new department, it is necessary to master the theoretical knowledge and operation of the equipment in the new department. Therefore, I feel stressed.” (N10).

##### Departmental examination

Nursing students undergo an examination at the end of their practicum in each department to test the effectiveness of their practicum, including a theoretical examination, an operational examination, and a nursing and teaching round examination. Nursing students need to spend much time preparing for these exams, which is undoubtedly stressful for them. In this study, four nursing students were stressed about these departmental examinations.

“Although the departmental examinations are not difficult, I still feel great pressure because the examinations involve many aspects, such as the theory examination, operation examination, nursing and teaching round examination.” (N6).

“Departmental examination is also very stressful for us because we have to prepare for lectures, nursing and the teaching round examination.” (N7).

##### Nurse certification examination

Nurse certification is a necessary reference for hospitals when hiring nurses, so it is vital for every nursing student. In this study, four nursing students were stressed about the nurse certification examination.

“The nursing certification examination is coming soon, but I have only prepared a little bit.” (N3).

“We have a nurse certification examination soon, but we know little about this examination.” (N5).

##### Graduation thesis

A graduation thesis represents an important step that nursing students must go through before graduation. However, nursing students have limited scientific research experience, which means that it is difficult for them to write such a thesis. As a result, graduation theses put a great deal of stress on nursing students. In this study, two nursing students were stressed about graduation thesis.

“For the graduation thesis, although we have teachers in the hospital to guide us, we do not feel that we get much help.” (N4).

“I am not particularly at ease because the graduation thesis issue has not been solved.” (N5).

##### School theory examination

The school regularly organizes theoretical examinations to help nursing students pay attention to theoretical studies in the clinical practicum. However, nursing students have limited time to read books. Conflict between study and work increases the psychological pressure experienced by nursing students. In this study, two nursing students were stressed about the school theory examination.

“During my clinical practicum, I am always busy with my work and don’t have time to read books, so it is difficult for me to cope with those exams.” (N2).

“During the clinical practicum, there are many theoretical examinations given by the school, but we have limited time to read books. So, the school theory examination is a little bit difficult for us.” (N9).

#### Interpersonal relationships

The theme of interpersonal relationships included four subthemes: nurse‒patient relationships, teacher‒student relationships, classmate relationships and doctor‒nurse relationships.

##### Nurse‒patient relationships

Taking care of patients is the job of nursing students, but nursing students are sometimes not understood by patients. In this study, ten nursing students were stressed about the nurse‒patient relationships.

“It was just a blood sugar test. He said he didn’t need it, with a tough attitude, which made me feel sad.” (N1).

“Some patients are grumpy and swear at will, which makes me a little bit scared.” (N2).

“When the nurse failed to get a venipentesis for a child, the family came to the nurse’s desk to throw all kinds of things, which made me afraid.” (N8).

##### Teacher‒student relationships

In addition to the patients, nursing students spend most of their time with their teachers during the clinical practicum. Working in harmony with their teachers strongly affects the mood of nursing students. In this study, five nursing students stressed about the teacher‒student relationships.

“Some teachers are strict or say something critical, which makes me sad.” (N5).

“I think a good relationship with the teacher is very beneficial to the entire clinical practicum, but if the relationship is not good, it will make the clinical practicum a disaster.” (N6).

##### Classmate relationships

During the clinical practicum, there is usually more than one nursing student in each department and dormitory, which leads to comparisons and contradictions between nursing students. In this study, two nursing students stressed about classmate relationships.

“At night, my roommates come back from work, and the noise affects my sleep. I can’t be very forceful about what others do; I can only mediate for myself.” (N4).

“If I compare myself with other students, I will feel that my operational proficiency is relatively weak, and then I will feel the pressure.” (N7).

##### Doctor‒nurse relationships

Although medicine and nursing are two independent disciplines, their work is closely related and inseparable. In this study, two nursing students said that doctors put a certain amount of pressure on them, especially in the operating room.

“Some doctors speak very softly and lose their temper easily. Then, I feel very stressed.” (N3).

“Those in the operating room pay close attention to their cooperation with the doctor. I feel a lot of pressure at that time.” (N5).

#### Occupational particularity

The theme of occupational particularity included five subthemes: work intensity, patient safety, specialized operation, occupational exposure and scheduling system.

##### Work intensity

Clinical nursing work is complicated and extends through the whole patient process from admission to discharge. This heavy workload causes nursing students to shuttle between wards all day, increasing their sense of fatigue. In this study, four nursing students experienced stress related to work intensity.

“The department is quite busy, and sometimes I feel tired.” (N2).

“I have to go back and forth every day. As time goes by, my legs, feet and back become obviously sore.” (N7).

##### Patient safety

Due to limited experience, nursing students feel powerless and stressed when encountering a variety of emergency incidents that threaten the safety of patients. In this study, six nursing students were stressed about patient safety.

“We failed to save the patient and then had to tell his family. When his family member saw him—it must have been his wife—she fell to the ground. I felt very touched inside and wanted to cry.” (N2).

“When the patient’s condition deteriorates and the teacher is not around, I don’t know who to turn for help, which is also very stressful for me.” (N8).

##### Specialized operation

Different departments have different specialized operations, with which nursing students are often not familiar. In this study, five nursing students were stressed about specialized operations.

“Now I am in the ICU. But I am not familiar with sputum sucking or ventilators, which makes me stressed.” (N5).

“When I perform unfamiliar nursing operations for patients, I feel very stressed.” (N8).

##### Occupational exposure

The clinical protection ability of nursing students is weak; thus, they might fail to take correct and effective protective measures in many cases, which can easily lead them to suffer from skin exposure, needle-stick injury and other conditions. In this study, six nursing students were concerned about their own safety.

“Because it is a department of infection, I am afraid of being infected. I worry about whether I will be stabbed by a needle. In addition, I also worry about my long-term contact with these patients.” (N4).

“The probability of needle stick injury is very high due to nursing work, which puts a certain amount of pressure on me.” (N9).

##### Scheduling system

Due to the particularity of the nursing profession, every nursing student must experience night shifts. In addition, some nursing students feel that the schedule is too tight, leading to a feeling of exhaustion. In this study, six nursing students were stressed about the scheduling system.

“Sometimes the schedule is too tight, and there’s not enough time for me to relax, so I feel tired.” (N2).

“The work is not regular and leads to various problems, such as physical discomfort, circadian rhythm disorders and endocrine disorders.” (N9).

#### Career planning

The theme of career planning included two subthemes: employment and further education.

##### Employment

The competition for jobs has become fierce in recent years. Nursing students are under great pressure to find jobs, especially when many students around them have already found jobs. In this study, five nursing students were stressed about employment.

“I think many students around me have already found jobs, so I have a little anxiety about my own employment.” (N6).

“In addition, I think the employment pressure is also very heavy because it is very difficult to find a job now.” (N8).

##### Further education

Some nursing students want to continue their studies by pursuing a master’s degree, which places great pressure on them. In this study, three nursing students were stressed about further education.

“For example, I have to prepare for the postgraduate entrance examination now. If I work in the meanwhile, it will be very difficult for me to allocate my time because I feel very tired from my work.” (N8).

“It is really difficult to take the postgraduate entrance examination this year. I’m not sure if I can go to my favorite school, so I feel a lot of pressure.” (N10).

### The themes and subthemes of coping styles

Two coping style themes were identified in this study: eliminating stressors and regulating emotions (Table 3).

**Table 3:** The themes and subthemes of coping styles

Themes	Subthemes	Number of students	Rank
Eliminating stressors	A. Improving self-ability	3 (N1, N3, N9)	4
	B. Sleeping	2 (N9, N10)	5
	C. Changing cognition	2 (N4, N8)	5
Regulating emotions	A. Going shopping	2 (N5, N10)	5
	B. Talking	9 (N1, N2, N3, N4, N5, N6, N7, N9, N10)	1
	C. Eating	6 (N1, N2, N4, N5, N6, N9)	2
	D. Entertainment	6 (N2, N4, N5, N7, N8, N10)	2

#### Eliminating stressors

The theme of eliminating stressors included three subthemes: improving self-ability, sleeping and changing cognition.

##### Improving self-ability

A large part of the pressure on nursing students comes from their own lack of theoretical knowledge and operational skills. Therefore, improving their self-ability is the key to effectively solving all kinds of pressure. In this study, three nursing students reported eliminating stress by improving their self-ability.

“Sharing the experience of caring for patients and knowing how to do better is a common way I handle stress.” (N1).

“Then, I come back to read more books, so I can be more familiar with the disease.” (N3).

##### Sleeping

Nursing students are required to work regular night shifts, which might lead to poor sleep quality. Faced with the stress of poor sleep, nursing students take advantage of sleeping to compensate for this stress. In this study, two nursing students eliminated stress by sleeping.

“I suffer from working irregularly and work hard, so I should sleep more.” (N9).

“The main way is to get more sleep because of the lack of sleep.” (N10).

##### Changing cognition

When facing the same stressful events, some students view such situations from a positive perspective, while others view them very negatively. Therefore, changing one’s cognition is an important way to eliminate stressors. In this study, two nursing students reported coping with pressure by changing their cognition.

“One thing is to think of things from a good direction. For example, although employment is very uncertain, there must be a job somewhere because there is a shortage of nurses. Thus, the salary must be relatively considerable.” (N4).

“If that doesn’t work, I’ll change some of my original practices or ideas.” (N8).

#### Regulating emotions

The theme of regulating emotions included four subthemes: going shopping, talking, eating and entertainment.

##### Going shopping

Shopping can help people relax and is an effective way to adjust their mood. In this study, two nursing students reported relieving their pressure through various forms of shopping.

“I will go out shopping and visit Taobao.” (N10).

“I usually like to go to the grocery store to pick up a few things.” (N5).

##### Talking

Talking is the most commonly used way to reduce stress by nursing students. In this study, nine nursing students reported talking to others to adjust their mood.

“I usually do it in the simplest way, which is to talk with my classmates or teachers.” (N6).

“If I can’t internalize it, I will to talk to my roommates, friends or family members. Their comforting words are also a good way for me to heal.” (N7).

##### Eating

Eating is one way for nursing students to cope with stress. Six nursing students said that eating could help them relieve pressure and better their mood, especially when eating dessert or a favorite food.

“Sometimes I might buy something delicious to give myself a treat.” (N2).

“Sometimes I eat something sweet or something I like to relieve my stress.” (N4).

##### Entertainment

Various forms of entertainment, such as listening to music, playing games, and watching TV, greatly enrich the lives of nursing students when they are under great pressure. In this study, six nursing students reported relieving their pressure through entertainment.

“I think playing games helps quite a lot.” (N5).

“I can watch interesting videos and listen to music. In addition, I like drawing very much. I always adopt these methods to relax myself.” (N7).

## Discussion

### Stressors of nursing students in the middle period of clinical practicum

The stress of nursing students is often related to personal reasons, such as financial stress and physical fitness. The nursing students in this study reported experiencing certain financial stress during their clinical practicum, which is consistent with previous research results [12]. The reason for this situation may be that nursing students spend a long time on their clinical practicum and are not paid during this period; they are responsible for their own daily expenses. In addition, physical fitness is also a stressor for the nursing students. Clinical work involves heavy tasks, and nursing students must have a strong body to cope with such work. Once nursing students have physical problems, their clinical work is affected [16], thus increasing their pressure.

Teaching arrangements, which include departmental rotation, departmental examination, nurse certification examination, graduation thesis and school theory examination, are common sources of stress for nursing students. Departmental rotation is a challenge for nursing students and increases their pressure. This may be related to the following reasons: (1) students may be afraid of making mistakes due to unfamiliarity with the equipment and care procedures in a new environment [13]; (2) students may be unacquainted with patients and teachers, which places them in a difficult position. In addition, departmental examination can also put pressure on nursing students. The potential reason for this situation may be that departmental examination not only increases the workload of nursing students but also leads them to worry about the examination results [14]. In addition, nurse certification examination is an additional stressor for nursing students. Nursing students are busy performing clinical work and thus have limited time to prepare for the nurse certification examination. The worry that failing the exam will affect their future work brings them great amount of additional pressure. Moreover, nursing students have to face pressure from not only the hospital at which they are engaged in their clinical practicum but also their school, such as through graduation thesis and school theory examination. This is consistent with the findings of a previous study [7]. On the one hand, thesis writing and theoretical examination account for a certain proportion of medical education, which directly affects graduation. On the other hand, reviewing theoretical knowledge and engaging in scientific research take considerable time, while nursing students’ energy is limited. Worrying about graduation and limited time puts considerable pressure on nursing students.

Interpersonal relationships are additional stressors of nursing students, according to our study. Nursing students need to address a variety of interpersonal relationships in the process of their of clinical practicum; the common relationships are nurse‒patient relationships, teacher‒student relationships, classmate relationships and doctor‒nurse relationships. The nurse‒patient relationship is a common stressor during the middle period of the clinical practicum. On the one hand, nursing students relatively lack operational skills and theoretical knowledge [31]. Patients and their families have a strong sense of distrust toward nursing students and often question their work or even refuse their care, which greatly increases their frustration. Some patients and their families are even prone to violent emotions due to illness or personality, which causes nursing students to worry about their personal safety. In addition, nursing students feel as though their teachers comprise one of the stressors during their clinical practicum, which is consistent with the findings of a previous study [15]. This may be due to the lack of consistency between students’ and teachers’ expectations [15]. Some teachers are strict, while means that some nursing students may not be able to meet the teacher’s requirements, which places additional stress on them. In addition, classmate relationships also put a great deal of pressure on nursing students. A comparison of operational ability and theoretical knowledge among students imperceptibly increases the stress of some nursing students [16]. Moreover, six students live together in a dormitory, and their irregular work and rest schedules may affect each other, which also places additional pressure on them. Furthermore, the nursing students herein expressed that the doctors place additional pressure on them. This finding is consistent with that of a previous study [11]. This situation may be because nursing students are not proficient enough in nursing skills or do not know the habits of doctors; therefore, they cannot cooperate efficiently with doctors, which results in tension between doctors and nurses.

Our study revealed that occupational particularity, which includes work intensity, patient safety, specialized operation, occupational exposure and scheduling system, is one stressor of nursing students. The finding that work intensity is the stressor is consistent with the findings of previous studies [16, 20]. This may be because nursing students in the middle period of their clinical practicum do not fully adapt to their busy clinical work, and they cannot reasonably cope with the work intensity, resulting in increased pressure. At the same time, the students examined herein were undergoing their clinical practicum during the COVID-19 pandemic, when the number of hospitalized patients was greatly increased, which would have further aggravated the work intensity of the students. Such increases also lead to increased stress in the nursing students. In addition, emergencies related to patient safety, such as accidental extubation, falls, critical illness and even death, can also bring great pressure to nursing students. This finding is similar to that of a previous study [19]. This outcome may be because nursing students have limited experience and cannot handle these emergencies well, which causes great pressure on them. Furthermore, specialized operations pressure the nursing students to a certain extent, which is consistent with the findings of one previous study [1]. Despite several months of clinical experience, some nursing students might not have the opportunity to be exposed to certain special operations or might be stressed because of the long interval since their last exposure. In addition, we find that occupational exposure is an important stressor for nursing students, as it may increase their infection risk. One previous study revealed that 70% of nurses suffer significant stress from exposure to needle stick injuries [32]. It is worth noting that this study was conducted during the COVID-19 pandemic; thus, the nursing students examined herein were more worried about becoming infected with the virus. Besides, the scheduling system is a common stressor for nursing students, which is similar to the findings of a previous study [17]. Night shifts and tight schedules are the most common problems in scheduling systems; these problems not only affect the concentration and work performance of nursing students but also negatively affect their health and ultimately lead to increased stress.

Career planning is also one of the stressors experienced by nursing students; it includes two subthemes: employment and further education. Employment is an important source of stress for nursing students, which is consistent with previous studies [7, 11, 18]. The reason may be that the number of nursing graduates has increased in recent years, while the core competitiveness of nursing undergraduates has declined [18]. Moreover, compared with previous years, the number of nursing positions has decreased. The two abovementioned reasons lead to increased employment pressure for nursing students. In addition, further education is also an important source of stress for nursing students, which is in line with the findings of a previous study [11]. Some nursing students choose to pursue a master’s degree, which means that they need to spend much time preparing for postgraduate entrance examination and face fierce competition for admission, resulting in great pressure.

### Coping styles of nursing students in the middle period of clinical practicum

Eliminating stressors is a common coping style for nursing students and includes three subthemes: improving self-ability, sleeping and changing cognition. Nursing students choose to improve their self-ability and sleep more to address stressors. This finding is consistent with that of Ahmed et al.’s study, which indicated that solving stressful events and sleeping more are approaches significantly utilized by nursing students to manage stressors [23]. If inadequate ability and sleep problems are important stressors for nursing students, then improving their self-ability level and sleeping ability are the keys to effectively addressing such stressors. It is worth noting that changing cognition is one of the coping styles of nursing students discussed herein, which is in line with the findings of a previous study [23]. This may be due to the important role that the cognitive system plays in assessing stressors and choosing coping styles; furthermore, examining problems with an optimistic attitude is an effective way to reduce stress.

In this study, nursing students reported regulating emotions to relieve stress, which included the subthemes of going shopping, eating, talking and entertainment. Nursing students consider shopping as a way to deal with pressure. Studies have shown that there is a close relationship between reducing stress and shopping [33], which is likely because shopping can regulate negative emotions to some extent. In addition, this study found that talking is a strategy used by nursing students to relieve stress, which is in line with the findings of previous studies [16, 26]. Sharing experiences with family members, classmates and other people around them through talking is a simple and effective way for these students to regulate their emotions. Nursing students can not only vent their emotions but also receive suggestions and psychological support while talking [26]. Besides, nursing students also choose to eat sweets or their favorite foods when faced with stress. This finding is consistent with one previous study, which noted that women prefer to eat sweets when experiencing stress [34]. This may be because eating has an effect on one’s brain function, which in turn regulates one’s emotions [35]. Furthermore, this study found that entertainment (such as listening to music and playing games) is also a way for nursing students to cope with stress. This finding is supported by previous studies that have reported that music therapy can promote emotional stability [36] and that games can significantly improve the emotional management of nurses [37]. This may be because entertainment can provide pleasant experiences for people, thus increasing their enjoyment.

### Comparison of stressors and coping styles between nursing students in the middle period of their clinical practicum and those in the early and late periods

The stressors experienced by nursing students in the middle period of their clinical practicum were different from those experienced in the early and late periods. A lack of professional knowledge or clinical skills, interpersonal relationships, clinical environment, patient care and workload are considered common stressors of nursing students in the early period of their clinical practicum [1, 19]. In addition, studies have shown that the pressure placed on nursing students in the late period of their clinical practicum mainly includes graduation requirements and employment [7]. In addition to the stressors experienced in the early and late periods of clinical practicum, nursing students in the middle period of their clinical practicum also experience special stressors, such as financial stress, physical fitness and further education. The possible reasons for these special stressors being present in the current study are as follows: (1) most of the nursing students in this study were from rural areas with relatively poor economic conditions, which could lead to more financial stress; (2) the nursing students in this study were all female and may have relatively poor physical fitness [38]; and (3) the nursing students who wanted to pursue a master’s degree need to spend much time preparing for the postgraduate entrance examination at this stage, and the enormous conflict between study and work time could cause great pressure to be placed on them.

With respect to coping styles, changing cognition, talking, and comforting oneself are the common coping styles used by nursing students in the early period of their clinical practicum [1]. In the late period of their clinical practicum, talking, comforting oneself and avoidance are the main coping styles used by nursing students to cope with stress [26]. In contrast to those used in the early and late periods of their clinical practicum, nursing students in the middle period of their clinical practicum adopt methods of improving self-ability, sleeping, going shopping, eating and entertainment to cope with stress. The reasons for using these coping styles are as follows: (1) nursing students’ need for respect and self-realization are prominent, and their clinical practice ability is still insufficient in the middle period of their clinical practicum, which means that improving their own ability is an effective way for them to cope with stress; (2) the number of night shifts increases in the middle period of the clinical practicum, which means that sleeping is an effective way to relieve stress; (3) the nursing students in this study were all female and may have simply preferred shopping and eating [39, 40]; and (4) nursing students experience many stressors in the middle of their clinical practicum, which means that various forms of entertainment may effectively reduce their level of pressure. It is worth noting that talking is an effective coping style for nursing students during different periods, which indicates that talking can effectively relieve the stress of nursing students at any time. Therefore, talking is an important coping style worth promoting for nursing students.

### Implications

The stressors and coping styles of nursing students in the middle period of their clinical practicum are different from those in other periods; this finding has important implications for nursing education. The following specific suggestions regarding the stressors of nursing students should be taken. (1) For personal reasons related to financial stress and physical fitness, nursing students should be encouraged to apply for grants from school and increase the level of physical activity in their daily life. (2) For teaching arrangements, schools should strengthen ties with hospitals to rationalize the arrangement of various examinations and departmental rotations. In addition, it is necessary to provide students with information about the nurse certification examination and experienced thesis advisors. (3) For interpersonal relationships, schools need to offer courses on interpersonal communication for nursing students. It is also a good idea to ask experienced instructors to share their interpersonal experience with nursing students. (4) For occupational particularity, schools can arrange for nursing students to go to a clinic during their theoretical study period to help familiarize them with and adapt to clinical nursing work in advance. (5) For career planning, schools should organize regular lectures on employment and further education for nursing students. Specific suggestions regarding the coping styles of nursing students are listed as follows. (1) For eliminating stressors, schools should strengthen the training offered for clinical skills and provide a comfortable sleeping environment. Moreover, it is necessary to cultivate positive cognition about the clinical work of nursing students through relevant lectures and courses. (2) For regulating emotions, schools can offer platforms for nursing students to seek peer support and advice. In addition, nursing students should be encouraged to relax themselves by shopping, eating and using various forms of entertainment.

### Limitations

Several limitations of this study should be noted. First, the participants came from only one hospital in China. Nursing students in other countries may have different experiences of stressors and coping styles during their clinical practicum. Second, all participants were women, which may have led to certain deviations in the research results. Third, quantitative measures were not used in this study, which means that quantitative information on the stress levels and coping styles of nursing students was not provided. Finally, this study explored only the stressors and coping styles of nursing students in the middle period of their clinical practicum and thus could not identify dynamic changes in the stressors and coping styles of nursing students during different periods.

## Conclusion

Nursing students experienced various stressors and used a variety of coping styles in the middle period of their clinical practicum, which was different from what occurred in the early and late periods. Targeted interventions should be formulated and implemented to relieve nursing students’ stress and guide them to adopt effective coping styles.

## Data Availability

The datasets used and/or analysed during the current study are available from the corresponding author on reasonable request.

## References

[1] Liu J, Yang Y, Chen J, Zhang Y, Zeng Y, Li J (2022). Stress and coping styles among nursing students during the initial period of the clinical practicum: a cross-section study. Int J Nurs Sci.

[2] Admi H, Moshe-Eilon Y, Sharon D, Mann M (2018). Nursing students’ stress and satisfaction in clinical practice along different stages: a cross-sectional study. Nurse Educ Today.

[3] Bartlett ML, Taylor H, Nelson JD (2016). Comparison of Mental Health Characteristics and stress between baccalaureate nursing students and non-nursing students. J NURS EDUC.

[4] Khamisa N, Peltzer K, Ilic D, Oldenburg B (2016). Work related stress, burnout, job satisfaction and general health of nurses: a follow-up study. INT J NURS PRACT.

[5] Luo ZY (1998). Psychological characteristics and counseling of nursing students in different periods of clinical practicum. Secondary Med Educ.

[6] Bhurtun HD, Turunen H, Estola M, Saaranen T (2021). Changes in stress levels and coping strategies among Finnish nursing students. NURSE EDUC PRACT.

[7] Deng XM, Cheng L, Chen K (2021). A qualitative study of factors related to psychological stress among nursing students in the late period of clinical practicum. Contemp Nurse (Upper Edition).

[8] Ma H, Zou JM, Zhong Y, Li J, He JQ (2022). Perceived stress, coping style and burnout of Chinese nursing students in late-stage clinical practice: a cross-sectional study. NURSE EDUC PRACT.

[9] Lazarus RS, Folkman S (1987). Transactional theory and research on emotions and coping. EUR J PERSONALITY.

[10] Labrague LJ, McEnroe-Petitte DM, Gloe D, Thomas L, Papathanasiou IV, Tsaras K (2017). A literature review on stress and coping strategies in nursing students. J MENT HEALTH.

[11] Liu XQ, Xu Z, Wang X, Zhang WJ, Xu YH, Liu WH, Liu H (2020). An applied study on factors related to psychological stressors and self-efficacy of clinical nursing students. China Health Ind.

[12] McCarthy B, Trace A, O’Donovan M, Brady-Nevin C, Murphy M, O’Shea M, O’Regan P (2018). Nursing and midwifery students’ stress and coping during their undergraduate education programmes: an integrative review. Nurse Educ Today.

[13] Aljohani W, Banakhar M, Sharif L, Alsaggaf F, Felemban O, Wright R (2021). Sources of stress among Saudi Arabian nursing students: a cross-sectional study. Int J Environ Res Public Health.

[14] Gazzaz ZJ, Baig M, Al AB, Al SM, Al AA, Al-Grad M, Qurayshah M (2018). Perceived stress, reasons for and sources of stress among medical students at Rabigh Medical College, King Abdulaziz University, Jeddah, Saudi Arabia. BMC MED EDUC.

[15] Al-Zayyat AS, Al-Gamal E (2014). Perceived stress and coping strategies among Jordanian nursing students during clinical practice in psychiatric/mental health courses. Int J Ment Health Nurs.

[16] Ching S, Cheung K, Hegney D, Rees CS (2020). Stressors and coping of nursing students in clinical placement: a qualitative study contextualizing their resilience and burnout. NURSE EDUC PRACT.

[17] Postma J, Tuell E, James L, Graves JM, Butterfield P (2017). Nursing students’ perceptions of the transition to Shift Work: a total Worker Health Perspective. WORKPLACE HEALTH SAF.

[18] Zhang CJ, Yi QF (2020). Advances in research on employment stress among nursing students. Contemp Nurse (Upper Edition).

[19] Li YJ, Shi TY, Lu J, Li H (2015). A qualitative study of undergraduate nursing students’ experience of stress in the early period of clinical practicum. Liberation Army Nurs J.

[20] Shaban IA, Khater WA, Akhu-Zaheya LM (2012). Undergraduate nursing students’ stress sources and coping behaviours during their initial period of clinical training: a Jordanian perspective. NURSE EDUC PRACT.

[21] Duan M, Zhang Y (2014). A qualitative study of the psychological experiences of undergraduate nursing students in the late period of clinical practicum. J NURS MANAGE.

[22] Sheu S, Lin HS, Hwang SL (2002). Perceived stress and physio-psycho-social status of nursing students during their initial period of clinical practice: the effect of coping behaviors. INT J NURS STUD.

[23] Ahmed W, Mohammed B (2019). Nursing students’ stress and coping strategies during clinical training in KSA. J Taibah Univ Med Sci.

[24] Zhang Y, Zhang H, Zhao S, Zhang L (2011). A quantitative and qualitative study of stressors and coping styles of nursing students during different periods of clinical practicum. NURS RES.

[25] Li XC, Liu CF, Yang TT, Li J (2022). A survey and analysis of nursing students’ stressors and their coping styles in the late period of clinical practicum. J Bengbu Med Coll.

[26] Zhang CP (2013). A qualitative study of psychological stressors and coping styles of nursing students in the late period of clinical practicum. Anhui Med.

[27] Huang GF. From description to interpretation: the Phenomenological Research path Shift. Social Sci 2017(10):52–6.

[28] Willis DG, Sullivan-Bolyai S, Knafl K, Cohen MZ (2016). Distinguishing features and similarities between descriptive phenomenological and qualitative description research. West J Nurs Res.

[29] Sun YL, Luo LH (2002). Study on Education Research Sampling ——Purpose sampling. J Yunnan Normal Univ (Philosophy Social Sci Edition).

[30] Liu M (2019). Application of Colaizzi’s seven steps in the analysis of phenomenological research data. J Nurs.

[31] Suikkala A, Leino-Kilpi H, Katajisto J, Koskinen S (2020). Nursing student-patient relationship and related factors-A self-assessment by nursing students. J CLIN NURS.

[32] Ali G, Mohamed M (2018). Evaluations of stress level caused by fear of exposure to needle stick Injury among nurses: a cross-sectional study. Tanta Sci Nurs J.

[33] Georgiadou E, Koopmann A, Müller A, Leménager T, Hillemacher T, Kiefer F (2021). Who was Shopping more during the Spring Lockdown 2020 in Germany?. FRONT PSYCHIATRY.

[34] Kim JG, Lee J, Song K (2021). Relationship between sweet food intake and stress among college students in Seoul and Gyeonggi areas. J Nutr Health.

[35] Weltens N, Zhao D, Van Oudenhove L (2014). Where is the comfort in comfort foods? Mechanisms linking fat signaling, reward, and emotion. Neurogastroenterol Motil.

[36] Meng JX. A study of the efficacy of Orff music therapy on social interaction, mood and attention in children on the autism spectrum. *Master* Wuhan Conservatory of Music; 2020.

[37] Zhang Y, Ma YM, Cao W (2020). The effect of group sandplay on the emotional management of surgical oncology nurses. Clin Med Res Pract.

[38] Yang ML, Xu XQ, Ma XM, Lou XM, Wang JJ, Yan GL, Wang Y, Liu DC (2022). Analysis on the status and related factors of physical quality among primary and middle school students in Henan Prov-Ince. Chin J School Health.

[39] Huang Y (2019). Battle of the sexes - a study of consumer gender differences and online shopping behavior. Natl Circulation Econ.

[40] Sun L, Luo D, Gao L (2022). Gender differences in nutrition awareness and dietary lifestyle among students from a college in Heze City. Occupation Health.

